# Direct Electrochemical Capture and Release of Carbon Dioxide Using an Industrial Organic Pigment: Quinacridone**

**DOI:** 10.1002/anie.201403618

**Published:** 2014-05-21

**Authors:** Dogukan Hazar Apaydin, Eric Daniel Głowacki, Engelbert Portenkirchner, Niyazi Serdar Sariciftci

**Affiliations:** Linz Institute for Organic Solar Cells (LIOS), Physical ChemistryAltenberger Strasse 69, 4040 Linz (Austria)

**Keywords:** carbon dioxide capture, electrochemistry, organic pigments, vat dyes

## Abstract

Limiting anthropogenic carbon dioxide emissions constitutes a major issue faced by scientists today. Herein we report an efficient way of controlled capture and release of carbon dioxide using nature inspired, cheap, abundant and non-toxic, industrial pigment namely, quinacridone. An electrochemically reduced electrode consisting of a quinacridone thin film (ca. 100 nm thick)on an ITO support forms a quinacridone carbonate salt. The captured CO_2_ can be released by electrochemical oxidation. The amount of captured CO_2_ was quantified by FT-IR. The uptake value for electrochemical release process was 4.61 mmol g^−1^. This value is among the highest reported uptake efficiencies for electrochemical CO_2_ capture. For comparison, the state-of-the-art aqueous amine industrial capture process has an uptake efficiency of ca. 8 mmol g^−1^.

The potential problems of climate change caused by anthropogenic carbon dioxide emissions constitute major issues faced by scientists today.[1] Technologies aim at capturing CO_2_, followed by sequestration or utilization, for example by reduction to useful hydrocarbons, such as formic acid,[2] carbon monoxide,[3,4] methanol,[5,6] or methane.[7] A key step for both sequestration and utilization of CO_2_ is controlled capture, storage, and release. The most efficient and widely used method in industry is post-combustion capture using amines. In this method, flue gas is pumped into a receiver which chemically captures and separates most of the CO_2_ from flue gas. For this purpose, an aqueous alkaline solvent or various amines are used as the capturing agents. After capture, the CO_2_-rich solvent is transferred to a separate compartment and heated to elevated temperatures for release of CO_2_ as gas and regeneration of the free solvent along with degradation and corrosion products. This process is energy intensive as a result of the temperature-swing process, and the volatility of the amine compounds leads to problems with their emission as air pollutants.[1] Several carbon-based solid-state materials have been explored as possible alternatives to the amine capture process, featuring various forms of porous graphitic materials[8–11] or microporous polymeric resins.[12–14] Appel et al.[15] introduced a capture and release method using Cu^+^/Cu^+2^ complexes which was expanded upon using an electrochemically mediated amine-based procedure with the advantage of operating at low temperatures.[16] This approach relies on the electrochemical regeneration of free amines in a Cu/Cu^+2^ redox process. The idea of direct electrochemical CO_2_ capture exploiting the redox behavior of molecular materials, however, remains largely unexplored. Some examples of redox active organic molecules reacting with CO_2_ can be found in the literature: A 1971 study by Reddy et al. found that electrochemically reduced benzalaniline reacts at 140 °C with CO_2_ to form 1-α-phenyl-phenylglycine.[17] In 1984, Sasaki et al. reported the electrochemical carboxylation of α,β-unsaturated ketones with carbon dioxide.[18] DuBois and co-workers showed the potential of electroactive CO_2_ carriers for capturing CO_2_ for space applications[19–21] Wrighton and Mizen studied similar quinone structures showing that CO_2_ undergoes reductive addition to chemically reduced 9,10-phenanthrenequinone, forming a biscarbonate dianion.[22] Later Stern et al. conducted theoretical calculations on 1,4-benzoquinone structures to support the mechanism proposed by Wrighton and Mizen.[23] Herein we conduct an analogous electrochemical reaction using a solid organic-pigment thin film, combining the advantages of electrochemistry with those of an organic solid active material. With this method, we were able to achieve CO_2_ uptake values on par with amine-mediated capture and exceeding most reported carbon-based methods. To our knowledge, this is the only process reported working with such an efficiency level while operating entirely at room temperature.

The method demonstrated herein relies on quinacridone (QNC), a common mass-produced industrial organic pigment, which we found can function to electrochemically capture and release CO_2_ with remarkable efficiency. Quinacridones (QNCs) belong to the extensive family of carbonyl dyes and pigments.[24,25] The carbonyl functional group which gives the family its name accounts for the well-known electron-accepting properties of these materials.[25] It is exactly this property that is exploited in the process of vat dying, practiced for thousands of years, whereby insoluble pigments are reduced to form water-soluble anionic dyes which readily penetrate into fabrics. QNC and similar materials are known to be stable organic semiconductors as well.[26]

QNC films (100 nm thickness) deposited on indium tin oxide (ITO) coated glass slides are electrochemically reduced in an acetonitrile electrolyte solution. We found that the introduction of CO_2_ gas into the solution results in a reaction with the reduced QNC pigment, forming a QNC⋅carbonate anion with tetrabutylammonium (TBA) acting as the counterion, according to Figure 1 a. Based on the experiments discussed herein, we propose the mechanism shown in Figure 1 b.

**Figure 1:**
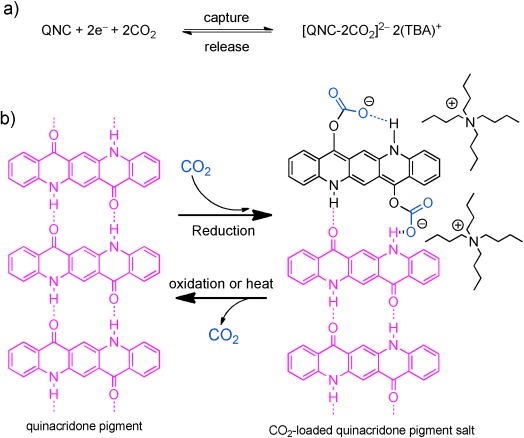
a) Electrochemical formation of the QNC-dicarbonate salt. b) Proposed mechanism for electrochemical capture and release of CO_2_ using the QNC pigment. Hydrogen-bond stabilized carbonate salts are formed in the film.

CO_2_-loaded QNC films are stable over several hours in ambient conditions. The trapped CO_2_ can be released either by electrochemically oxidizing or heating the film. In all cases, Fourier transform infrared (FT-IR) measurements were used to quantify the amounts of captured and released CO_2_. We found that 20 wt % capture (20 g CO_2_/100 g QNC) is possible using this reduce-and-capture method, corresponding to CO_2_ uptake values of 2.28 mmol g^−1^ and 4.61 mmol g^−1^ in thermal and electrochemical cases, respectively. This figure of merit considers the weight of the active CO_2_-capturing quinacridone film alone, excluding weight of the glass substrate and electrodes. Especially in the electrochemical release case, the obtained value is higher than most of the literature values[8,11–13] and very close to the uptake efficiencies achieved by activation at higher temperatures and high pressures.[10,14,27] For comparison, the established aqueous amine-based capture processes are the most efficient available, and have an approximately 8 mmol g^−1^ CO_2_ uptake.[28] Although the monoethanolamine system is applied industrially, it is not energy efficient owing to the high heat capacity of the sorbent solution in combination with the required temperature swing to regenerate the sorbent. The method demonstrated herein has about half the uptake efficiency, but the energy input is mild in comparison: CO_2_ can be released by heating, beginning at roughly 40 °C (Figure 2 a). CO_2_ can also be released by oxidation, using an overpotential of approximately 10 mV vs. Fc/Fc^+^ (Fc=[(η-C_5_H_5_)_2_Fe]; Figure 3). The processes are reversible and the same QNC films can be used over roughly five cycles efficiently, with the limiting factor being dissolution of quinacridone in the electrolyte over time.

**Figure 2:**
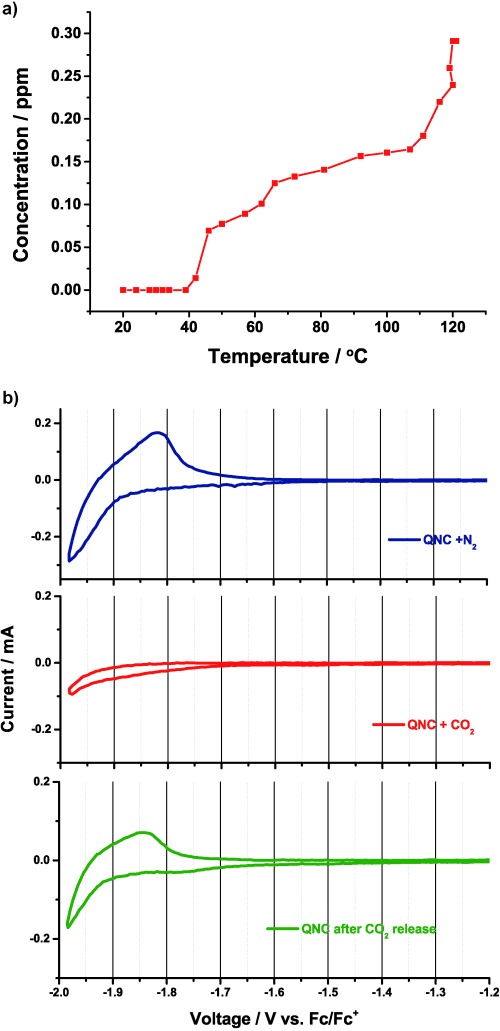
a) Amount of released CO_2_ by increasing temperature that is, decarboxylation b) CV of a QNC film under N_2_ (blue), under CO_2_ (red) during formation of the carbonate salt, and again under N_2_ (green) after thermal release of CO_2_.

**Figure 3:**
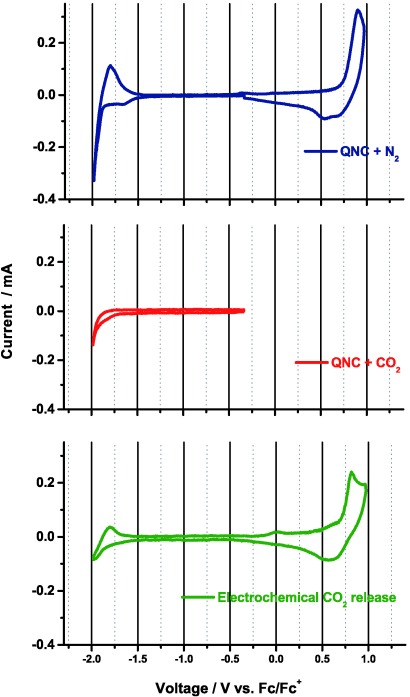
Cyclic voltammograms of QNC upon electrochemical capture and release of CO_2_.

For the capture of CO_2_ an electrochemical cell consisting of three electrodes: (ITO/QNC as working, Pt foil as counter, and Ag/AgCl (pseudo-reference which is calibrated against the Fc/Fc^+^ couple) was used. The cell was purged with N_2_ first and the QNC-coated ITO electrode was submerged into the solution. Cyclic voltammograms (Figure 2 b) were recorded in N_2_-purged and CO_2_-purged solutions. Upon purging with CO_2_ the characteristic reduction peak of QNC at −1820 mV diminished and the amount of cathodic current decreased (Figure 2 b). This phenomenon was related to the formation of a QNC⋅carbonate salt (Figure 1 b). To confirm that CO_2_ was effectively trapped in the film, the electrode was removed, washed with acetonitrile to remove any residual electrolyte salts, and placed into a sealed container with a gas-tight silicone septum on top. The sealed container was then connected to a FT-IR gas cell. The container containing the QNC film inside was placed on a hot plate. The temperature was increased gradually up to 120 °C resulting in decarboxylation, and the corresponding increase in the amount of CO_2_ was monitored by FT-IR absorption (Figure 2 a).

The concentration of released carbon dioxide was determined by calculating the area of the characteristic carbon dioxide peak at 2350 cm^−1^ and found to be 0.3 ppm. Furthermore, to confirm the release of CO_2_, the electrode was subjected to another cyclic voltammetry measurement after heating. The aforementioned cathodic peak which had diminished was observed to largely recover and electrochemical activity of QNC returned to the previous state (green curve, Figure 2 b). A control experiment of the same system and time without heat treatment did not yield any significant release of CO_2_, thus the quinacridone-CO_2_ adduct is relatively stable. Likewise, heating quinacridone films that had been stored under CO_2_ did not yield any CO_2_ capture/release—thus electrochemical reduction is necessary for reaction of CO_2_.

After reduction and capture, CO_2_ could also be released by electrochemically oxidizing the film. This was observed by mounting the electrochemical setup in a gas-tight vial. First, a full scan ranging between −1990 mV and 1010 mV vs. Fc/Fc^+^ was performed (Figure 3, blue curve), showing the characteristic reduction and oxidation peaks for QNC. Then the sealed vial was purged with CO_2_ for 20 min and QNC was reduced to capture the CO_2_ (Figure 3, red curve following capture). The vial was then purged extensively with N_2_ to avoid any remaining CO_2_ from the previous experiment. Scanning from 0 V towards positive potentials furnished a new peak with onset around 10 mV, followed by the characteristic oxidation peak of QNC at more positive voltages (Figure 3, green curve). Using a gas-tight syringe, a sample was taken from the headspace of the vial used throughout the experiment and injected to the gas-cell compartment of the FT-IR. The measurement confirmed that captured CO_2_ was released by electrochemical oxidation, starting at around 10 mV. Cyclic voltammograms recorded before, during, and after CO_2_ capture are shown in Figure 3. The amount of CO_2_ released upon oxidation was found to be 2 ppm.

The molar ratio between QNC and released CO_2_ was calculated for both thermal and electrochemical release cases. The amount of QNC in the film was calculated using the dimensions of a film (width, length, height: 0.9 cm, 3 cm, 1×10^−5^ cm) as 1.35×10^−7^ mol. For the thermal release case, the amount of CO_2_ was determined as 9.60×10^−8^ mol. Correspondingly, the released amount in the electrochemical case was found to be 1.94×10^−7^ mol. Molar ratios acting in CO_2_ capture were determined to be 0.70 and 1.43 for the thermal and electrochemical CO_2_ capture, respectively. In this respect it can be concluded that the electrochemical release is more effective than the thermal release. Next, based on these results we cannot definitively determine the stoichiometry of the reaction, that is, whether one or two carbonates attach to each QNC molecule, as the reaction is likely more complete at the surface of the film than in depth. Nevertheless it is likely that there are two carbonate groups per QNC molecule based on the 1.43:1 ratio found in the electrochemical release case, and also based on the assumed formation of a dienolate structure[25] in the QNC molecules. The carbonyl family of dyes and pigments are known to undergo electrochemical reduction owing to the electron-accepting properties of conjugated segments with carbonyl groups. Like the better-known indigoid and anthraquinone vat dyes,[24] QNC forms a dienolate structure upon two-electron reduction.[25] We propose that each enolate attacks a CO_2_ molecule, forming a dicarbonate structure that is stabilized by the extensive hydrogen bonding in the QNC solid state. The ultimate dicarbonate QNC carries a net −2 charge, with two tetrabutylammonium counterions. The formation of such a salt explains the disappearance of the cathodic behavior of QNC upon exposure to CO_2_ during electrochemical reduction. This mechanism was proposed for CO_2_ reaction with the quinone in the work of Wrighton and Mizen.[22]

A disadvantage of using vat dyes is that they are soluble in their reduced forms. Thus, with successive cycles of capture and release, the quinacridone film will dissolve. The capture–release process was subjected to a cyclic stability test over 15 cycles and the system showed a decrease in the amount of released CO_2_ with each subsequent cycle. The highest amount of captured carbon dioxide is released at first cycle. By the 15th cycle the process is no longer measurable. To see the effects of water, we conducted experiments in aqueous electrolytes. However, the required potentials to reduce quinacridone are out of the electrochemical window of aqueous solvents hence the process is superseded by H_2_ evolution in water.

This direct electrochemical capture of CO_2_ is a low-energy, low-temperature process and the combination of the mild capture/release conditions with a nontoxic and cheap industrial pigment make this route potentially advantageous for carbon capture and utilization processes. To overcome the issue of solubility of the reduced species in the electrolyte, we are currently working on polymeric carbonyl vat dyes, with the ultimate goal of stable electrochemistry in aqueous media. The ‘old’ chemistry of organic dyes and pigments may indeed contain interesting and unexplored potential solutions to modern problems.
