# Supplementary material for: Direct Electrochemical Capture and Release of Carbon Dioxide Using an Industrial Organic Pigment: Quinacridone
Source: Angew Chem Int Ed Engl. 2014 May 21;53(26):6819–22. doi: 10.1002/anie.201403618 (PMC4499243; doi:10.1002/anie.201403618)
Supplement: Supplementary file 1 [file anie0053-6819-sd1.pdf]

Angewandte Chemie
Eine Zeitschrift der Gesellschaft Deutscher Chemiker

Supporting Information
© Wiley-VCH 2014
69451 Weinheim, Germany

Direct Electrochemical Capture and Release of Carbon Dioxide Using an Industrial Organic Pigment: Quinacridone**

Dogukan Hazar Apaydin, Eric Daniel Głowacki, Engelbert Portenkirchner, and Niyazi Serdar Sariciftci*

anie_201403618_sm_miscellaneous_information.pdf

Supplementary Information File

Experimental Details:

QNC, obtained from TCI and purified by temperature gradient sublimation, was thermally evaporated onto ITO coated glass slides at a base pressure of a 10^{-6} mbar. Total thickness was 100 nm, verified by profilometry.

The electrochemical cell used for all experiments in shown in Figure S1. Glass/ITO/QNC functioned as the working electrode, a Pt foil was the counter electrode, while an Ag/AgCl wire was used as the quasi-reference electrode. Ferrocene was used as an external standard. The supporting electrolyte was 0.1M $TBAPF_6$ in anhydrous acetonitrile. All electrochemical measurements were conducted inside a N_2-filled glove box equipped with inlet for N_2 and CO_2 purging. Reduction of QNC films were conducted in a potential range from -340 to (-1990) mV with a scan rate of 50mV/s. For the electrochemical oxidation leading to the release of captured CO_2, carbon dioxide rich films were oxidized first and reduced subsequently in order to verify reversibility of the system. FT IR measurements were carried out on a Bruker IFS66S.

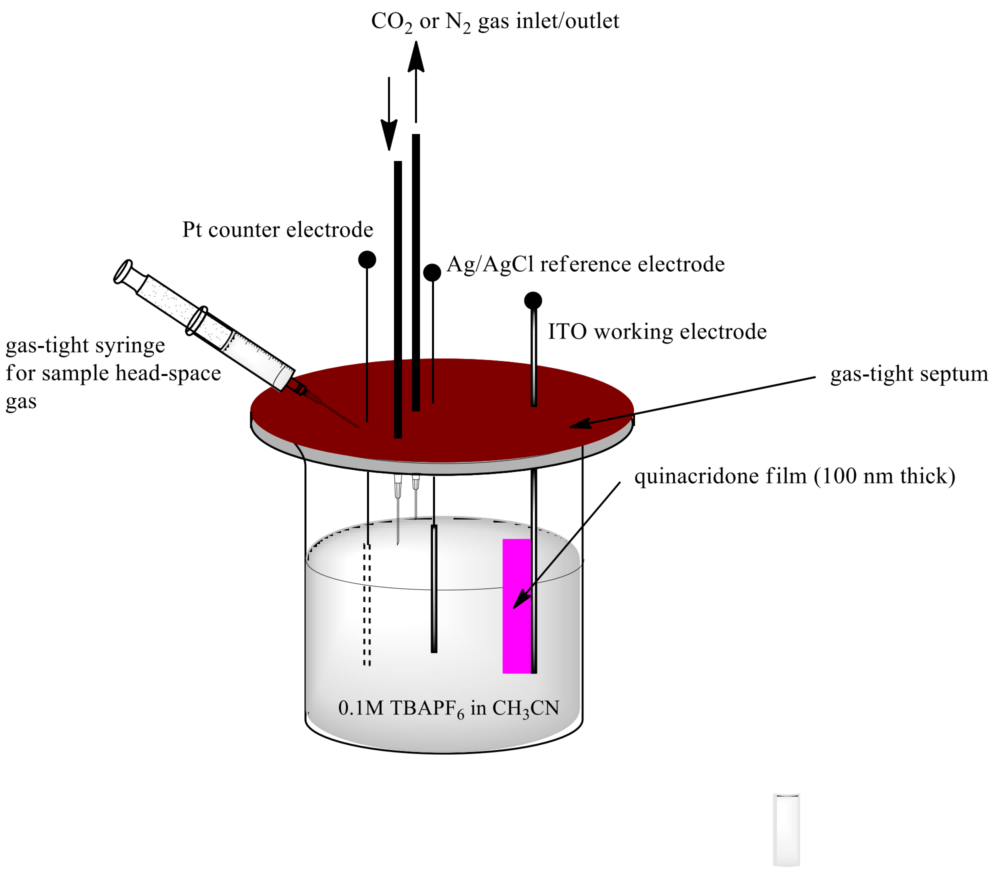

Figure S1. Electrochemical gas-tight cell used for all experiments
